# Free Microsurgical and Pedicled Flaps for Oncological Mandibular Reconstruction: Technical Aspects and Evaluation of Patient Comorbidities

**DOI:** 10.5402/2012/792674

**Published:** 2012-03-07

**Authors:** Victor J. Hassid, Suhair Maqusi, Emmett Culligan, Mimis N. Cohen, Anuja K. Antony

**Affiliations:** Division of Plastic, Reconstructive and Cosmetic Surgery, University of Illinois at Chicago, Chicago, IL 60612, USA

## Abstract

Oncologic mandibular reconstruction has changed significantly over the years and continues to evolve with the introduction of newer technologies and techniques. Patient demographic, reconstructive, and complication data were obtained from a prospectively maintained clinical database of patients who underwent head and neck reconstruction at our institution. The free fibular flap is now considered the gold standard for mandibular reconstruction. However, in patients with multiple comorbidities, lengthy procedures may be less optimal and pedicled flaps, with specific modifications, can yield reasonable outcomes. Technical aspects and comorbidity profiles are examined in the oncological mandibular reconstruction cohort.

## 1. Introduction

Oncologic mandibular reconstruction has changed significantly over the years and continues to evolve with the introduction of newer technologies and techniques. The goals of reconstruction, following oncologic resection, are both functional and aesthetic. Functional considerations include successful wound closure of the oropharynx, preservation of a patent upper airway, phonation, mastication, and potential for dental rehabilitation, in addition to restoration of aesthetic impairment. The principles that guide oncologic mandibular reconstruction focus on optimizing outcomes and identifying ideal flap reconstruction, with consideration of patient co-morbidities and reconstructive requirements.

Reconstruction with pedicled pectoralis major myocutaneous and deltopectoral flaps used to be the standard of care and continues to be in selected cases [1]. Free-flap reconstruction of oncologic defects has become the modern standard of care, largely due to superior functional and aesthetic outcomes [2]. However, in patients with multiple co-morbidities, who cannot tolerate lengthy surgery or fluid shifts, pedicled flaps may be best suited to meet the reconstructive requirements.

## 2. Materials and Methods

Patient demographic, reconstructive, and complication data were obtained from a prospectively maintained clinical database of patients who underwent head and neck reconstruction at the University of Illinois at Chicago Medical Center. Institutional Review Board approval was obtained. All patients who underwent oncological mandibular reconstruction were included in this study, representing a single surgeon's experience (A. K. Antony) from October of 2010 to May of 2011. Medical records were retrospectively reviewed to further characterize comorbid conditions and technical modifications employed to optimize results.

The following variables were reviewed: (a) underlying diagnosis, (b) age at diagnosis/surgery and gender, (c) operative procedures performed and technical characteristics, (d) pathological and clinical tumor stage (e) imaging studies (computerized tomography, X-rays), (f) co-morbidities and adjuvant therapy, (g) smoking history, (h) complications, and (i) functional and aesthetic outcome.

Follow-up of the patients was from 6 months to 1 year. Clinical examination was performed during regular clinic visits, in order to assess functional and aesthetic outcome, including dental occlusion and potential complications.

## 3. Results

Over the study period, 79 flap reconstructions (pedicled and free) were performed by a single surgeon. Twenty-three flaps for the head and neck were carried out in 20 patients. Of these, 12 patients required oncological mandibular reconstruction and were included in the study cohort. The age range of our patient population was 18 to 72 years old. Eleven of 12 patients underwent mandibular reconstruction after resection of underlying malignancy (9 had squamous cell carcinoma, and 2 mucoepidermoid carcinoma), and 1 patient underwent reconstruction after resection of ameloblastoma. All patients had immediate reconstruction, 7 with free fibula osteocutaneous flap, 1 with free vertical rectus myocutaneous flap, and 4 with pedicled pectoralis major myocutaneous flap. A reconstruction plate was used in all patients. Pathological staging was significant for T4 lesion (invasion of adjacent structures) in all malignant cases and 4 patients had N2b disease (multiple ipsilateral nodes, <6 cm). No flap losses were encountered (100% success rate).

All patients who underwent reconstruction with pedicled pectoralis major flaps had multiple (2 or more) co-morbidities and/or concomitant illness, which precluded lengthy surgery. These included poorly controlled diabetes mellitus (*n* = 4), chronic obstructive pulmonary disease (*n* = 1), aortic stenosis (*n* = 1), hepatocellular carcinoma (*n* = 1), hypertension (*n* = 1), end-stage renal disease on hemodialysis (*n* = 1) after failed renal transplant, and hyperlipidemia (*n* = 2). Two of 4 patients had positive drug (cocaine, *n* = 1) and/or tobacco (*n* = 2) use.

In the free flap reconstruction group (*n* = 7), 4 patients were otherwise healthy. Four patients had controlled hypertension; 2 patients had well-controlled diabetes mellitus; three patients had a remote smoking history; one patient was found to be actively smoking during the perioperative period; one patient had stable coronary artery disease and 1 hypothyroidism. Five of the six free fibula patients underwent medical modeling with excellent aesthetic and functional outcome.

All patients with malignant disease received either adjuvant (*n* = 10) or neoadjuvant (*n* = 1) radiation. Complications occurred in 2 patients in the free flap group. One patient, who underwent free fibula reconstruction for osteoradionecrosis, secondary to radiotherapy, continued to smoke in the preoperative period; this patient developed native skin necrosis in the postoperative period, requiring operative debridement and additional reconstruction with a pedicled pectoralis major myocutaneous flap. Another patient sustained skin radionecrosis after postoperative radiation and required wound debridement, plate removal, and reconstruction with a pedicled supraclavicular fasciocutaneous flap. Both patients recovered uneventfully, following their last reconstructive procedure.

## 4. Discussion

Reconstruction of mandibular defects is challenging, both technically and aesthetically. Pedicled flaps, such as the pectoralis major musculocutaneous flap, have traditionally served as the workhorse flaps for such reconstruction. The use of these flaps in the current era should be usually limited to patients whose medical co-morbidities preclude reconstruction with free tissue transfer, or to patients who have failed initial free flap reconstruction, as a salvage procedure. Pedicled flaps have traditionally endured suboptimal results, with a high incidence of complications after adjuvant therapy [1]. 

Microvascular free flap reconstruction after oncologic resection of head and neck cancers was popularized in the United States in the 1980s and 1990s [3]. Since then, free flap reconstruction of oncologic mandibular defects has been demonstrated to provide reliable results with high success and low morbidity rates [4]. For the majority of modern reconstructive plastic surgeons who perform mandibular reconstruction, vascularized composite-free tissue transfer, and specifically the free fibula flap, represents the gold-standard approach [5, 6]. Other flaps such as the ilium, scapula, and radius are mostly of historical interest, as the free fibula has emerged as the more ideal flap, with excellent bone quality and potential for multiple osteotomies [7]. The excellent vascularity, availability of a variety of tissue types (bone, muscle, skin), and ability to “custom fit” the defect in a single stage are among the advantages of free microvascular flaps for oncologic mandibular reconstruction. On the other hand, the need for high technical expertise, microsurgical specialized equipment, as well as optimal patient clinical status in order to withstand longer anesthesia, fluid shifts, and blood loss are among the disadvantages.

**Figure 1:**
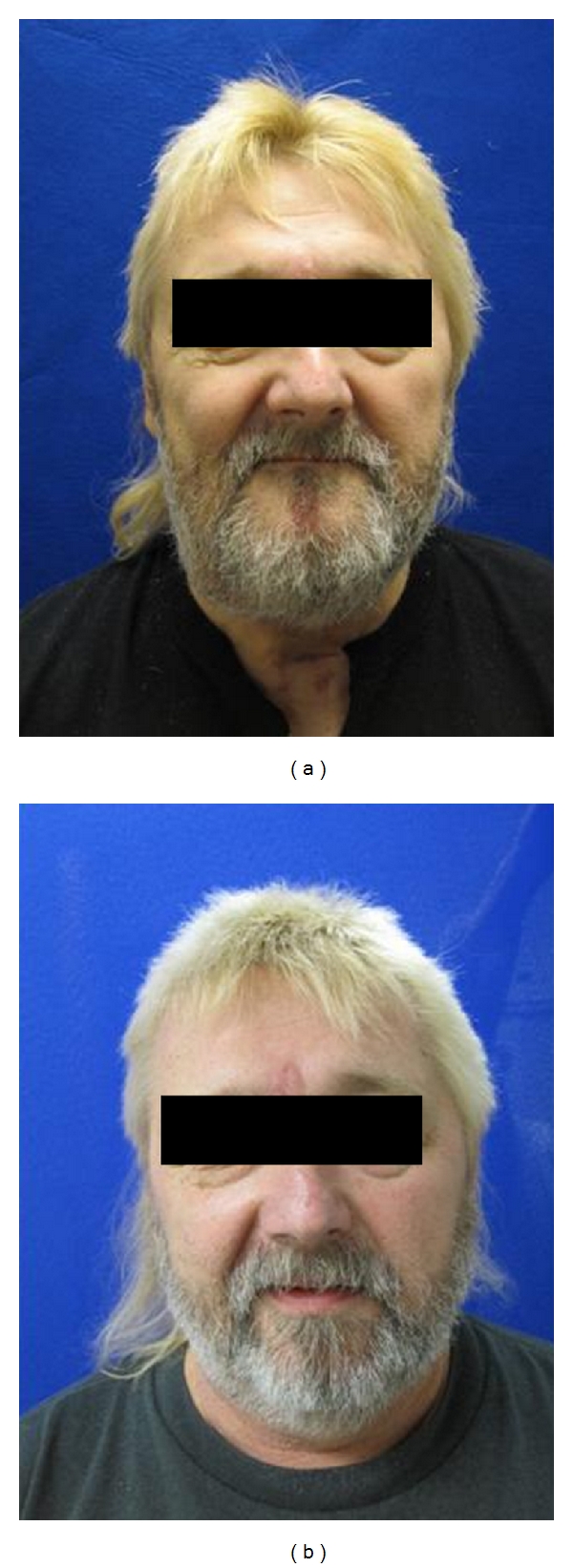
Preoperative (b) and Postoperative (1-year) (a) result after reconstruction with free fibula osteocutaneous flap for mandibular reconstruction.

**Figure 2:**
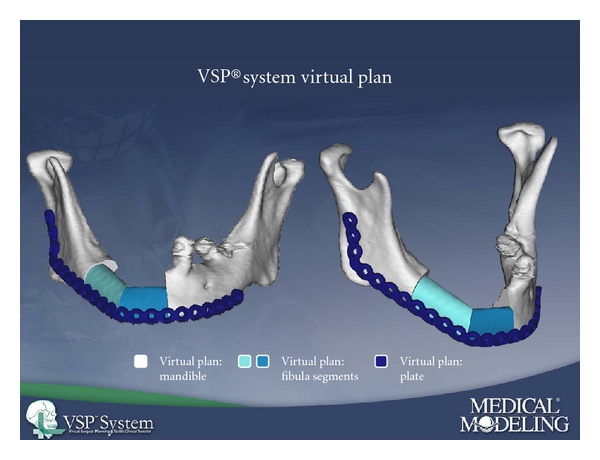
Virtually planned neomandible after fibular osteotomies for patient in Figure 1.

**Figure 3:**
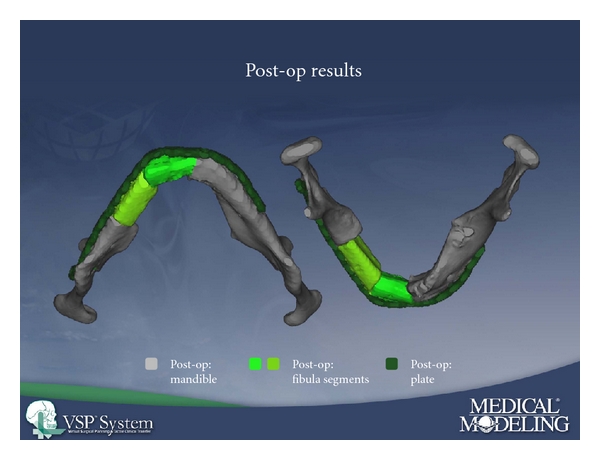
Postoperative neomandible with reconstructive plate in place.

The contouring of free fibula flap, although traditionally performed with a free-hand approach, is now being performed with more accuracy using preoperative virtual planning, which was the predominant technique employed in this study. Virtual planning allows for more accurate and efficient intraoperative fibular osteotomies and resultant neomandible, due to elimination of free-hand contouring [8]. Our preliminary experience with stereolithography-guided fibular osteotomies in mandibular reconstruction is favorable, and we believe that it will eventually become the standard of care [9] (Figures 1, 2, and 3).

In our study, we elected to proceed with pedicled reconstruction in patients with multiple co-morbidities, who were at higher risk for complications (Table 1). Use of pedicled regional flaps should be considered in cases of patients who would not tolerate lengthy free flap reconstruction of mandibular defects, as well as in cases of salvage reconstruction. Pedicled flap reconstruction is limited by the bulkiness of the flap, donor-site morbidity, and concerns over skin paddle reliability. In situations when the initial free flap reconstruction fails, the reconstructive surgeon has to determine the extent of failure (partial or total), the type and timing of intervention needed, and the potential for a second free flap versus a local or regional flap [1]. Two of our patients required a secondary regional pedicled flap (pedicled pectoralis major myocutaneous and supraclavicular fasciocutaneous flaps), since a second free flap was considered less desirable by either the patient or surgeon.

**Figure 4:**
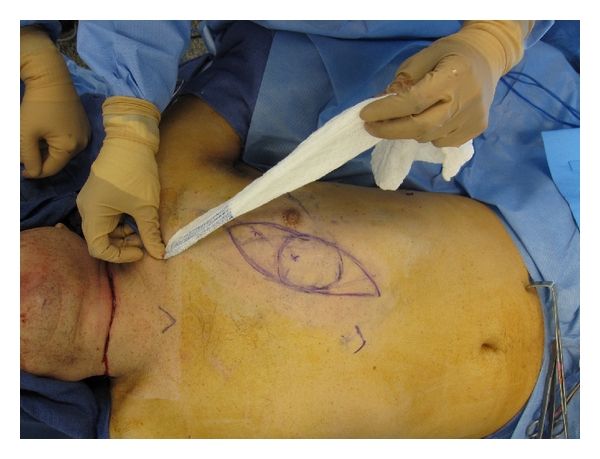
Template demonstrating modified musculocutaneous pectoralis major flap with planned arc of rotation.

**Figure 5:**
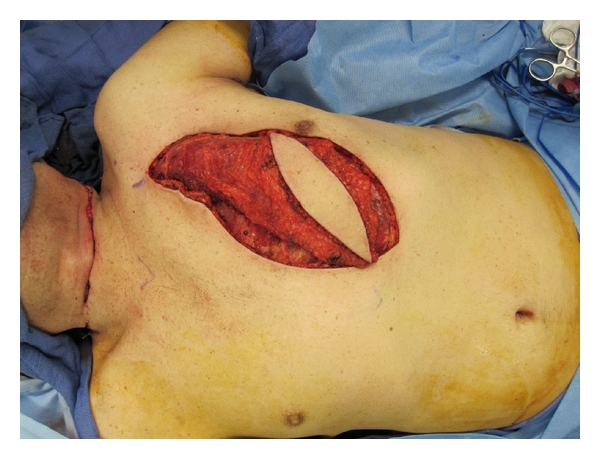
Pectoralis major musculocutaneous flap dissected.

Technical modifications of pedicled pectoralis flap include muscle thinning at the pedicle origin, planning of the skin island with Doppler ultrasound, and intraoperative planning of the arc of rotation with a template and judicious use of skin grafting in the restricted, radiated neck Figures 4 and 5. Additionally, consideration should be made to alternative pedicled flaps in secondary reconstruction in the female patient Figures 4 and 5. 

The reconstructive surgeon should also evaluate the role of adjuvant treatments, which have gained popularity in the treatment of head and neck malignancies. As radiation and chemotherapy treatment protocols are included with increased frequency in the treatment of mandibular malignancies, the reconstructive surgeon should be aware of the damaging effects of radiotherapy on local soft tissue and the overall wound healing process. If radiation has been used in a neoadjuvant setting, tissue away from the irradiated field should be used for reconstruction. If adjuvant radiotherapy is considered, the frequency of wound complications increases [10]. In our study, secondary reconstruction was required in two patients who received radiation therapy (adjuvant, neoadjuvant). Although the positive effects of radiation for the treatment of specific head and neck cancers are well established, it should be emphasized that it is not free of complications, such as skin radionecrosis, and direct communication between the reconstructive surgeon and the radiation-oncologist is encouraged in order to discuss the extent of the field that will be irradiated, as well as the total amount of radiation that will be used [11].

In regards to the presence of co-morbidities among the candidates for mandibular reconstruction, the association of tobacco use and increased complication rate following microvascular free tissue transfer is well established [12]. Although they are not necessarily associated with complete flap loss, smoking and diabetes have been linked to wound breakdown, hematoma formation, and prolongation of hospital course [12]. One of our patients who required a secondary pedicled flap for coverage, due to native skin necrosis, continued to smoke during the perioperative period, disregarding strong recommendations to abstain from tobacco use.

**Figure 6:**
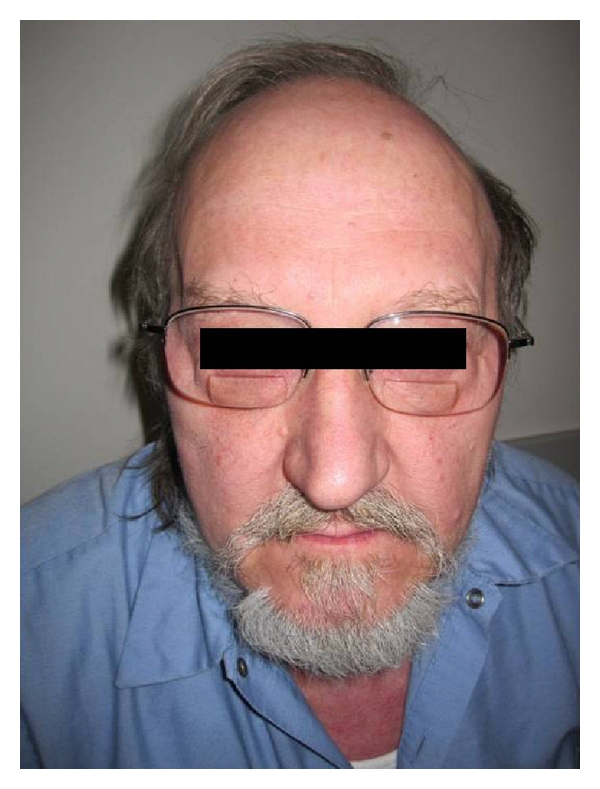
Preoperative picture.

**Figure 7:**
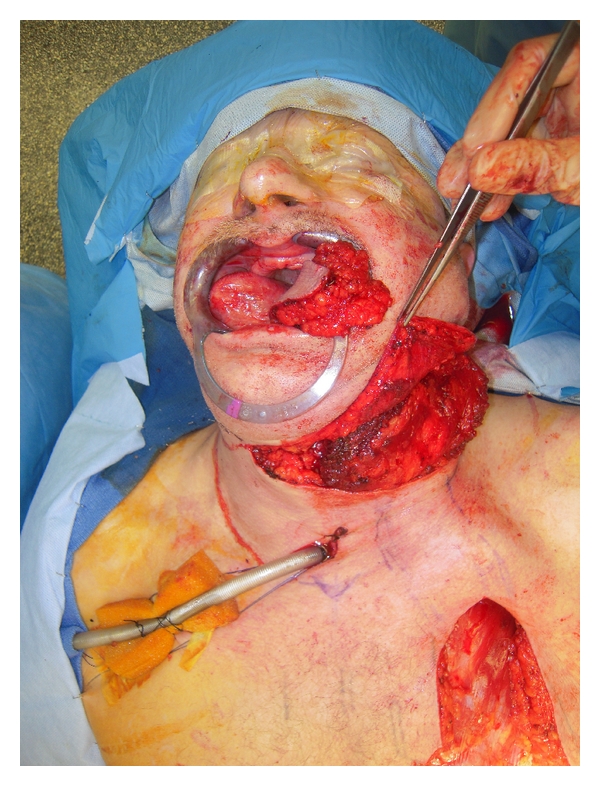
Intraoperative picture-Pectoralis major myocutaneous flap dissected and transposed into oral cavity.

**Figure 8:**
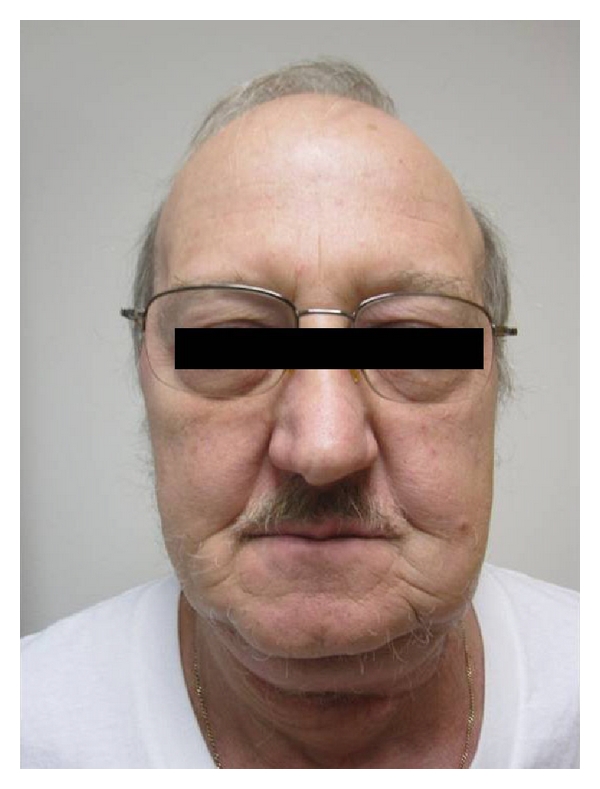
Postoperative picture.

Functional and aesthetic outcomes were excellent after use of free flap reconstruction. The customization of the free fibula, with virtually planned osteotomies to produce a well-fitted neomandible, contributes to an excellent functional and aesthetic result with high patient satisfaction [9]. Pedicled flaps, along with the modifications presented, can also yield excellent functional and aesthetic outcomes in patients with multiple co-morbidities (Figures 6, 7, and 8).

## 5. Conclusions

The free fibula flap is considered the dominant free flap for any oncologic mandibular reconstruction (except in cases of peroneus magnus) [13]. It represents high-quality bone with excellent length, which can tolerate multiple osteotomies and be contoured to replicate the mandibular shape. Emerging technologies, which have contributed to more accurate and efficient preoperative planning and shorter operative times, are gaining popularity and may eventually become the gold standard in mandibular reconstruction.

The role of pedicled flaps in oncologic mandibular reconstruction is significant and any reconstructive plastic surgeon should carefully assess the patient characteristics and the presence of co-morbidities. Technical modifications may be employed in order to provide the patient a more optimal reconstruction.

## Figures and Tables

**Table 1:** Pedicled and free flap reconstruction patient groups. PMMF*: *Pectoralis major myocutaneous flap; SCCA: squamous cell carcinoma; ESRD: end-stage renal disease; HL: hyperlipidemia; HD: hemodialysis; HTN: hypertension; DM: diabetes mellitus; COPD: chronic obstructive pulmonary disease; AS: aortic stenosis; HCC: hepatocellular carcinoma; CHF: congestive heart failure; CAD: coronary artery disease; XRT: radiotherapy; VRAM: vertical rectus abdominus myocutaneous flap.

	Age (yrs)	Gender	Procedure	Tumor type (pathological stage)	Radiation (Adj/Neo-adj)	Tobacco use (Yes/No)	Comorbidities (Complications/Treatment)
(1)	55	M	Pedicled PMMF	SCCA (T4aN2bM0)	A	No	ESRD on HD (failed renal transplant), DM, HTN, HL
(2)	57	F	Pedicled PMMF	SCCA (T4bN0Mo)	A	Yes	DM, hypercholesterolemia, Cocaine abuse
(3)	60	M	Pedicled PMMF	SCCA (T4aN0M0)	A	Yes	DM, COPD, AS
(4)	47	M	Pedicled PMMF	SCCA (T4N0M0)	A	No	DM, HCC, HL
(5)	56	F	Free fibula osteocutaneous flap	Mucoepidermoid carcinoma (T4aN0M0)	A	No	None (Skin radionecrosis from XRT; pedicled supraclavicular flap)
(6)	24	M	Free fibula osteocutaneous flap	Ameloblastoma	N/A	Quit 6 wks prior to surgery	None
(7)	19	F	Free fibula osteocutaneous flap	SCCA (T4aN2b,M0)	A	No	None
(8)	64	M	Free fibula osteocutaneous flap	SCCA (T4a,N0M0)	A	Quit 8 yrs prior to surgery	DM, CHF, HTN
(9)	62	M	Free fibula osteocutaneous flap	SCCA (T4aN0M0)	A	No	renal transplant, HTN, stable CAD, HL
(10)	60	F	Free fibula osteocutaneous flap	SCCA (T4aN2bM0)	A	No	HTN, hypothyroidism, HL
(11)	73	F	Free fibula osteocutaneous flap	SCCA (T4N0M0) Osteoradionecrosis (XRT)	N	Yes	DM, HTN. (Native skin necrosis due to perioperative active smoking, h/o XRT; pedicled PMMF)
(12)	47	M	Free VRAM flap	SCCA (T4aN2bM0)	A	Quit 4 wks prior to surgery	None
